# cRNAsp12 Web Server for the Prediction of Circular RNA Secondary Structures and Stabilities

**DOI:** 10.3390/ijms24043822

**Published:** 2023-02-14

**Authors:** Fengfei Wang, Wei Li, Baiyi Li, Liangxu Xie, Yunguang Tong, Xiaojun Xu

**Affiliations:** 1 Institute of Bioinformatics and Medical Engineering, Jiangsu University of Technology, Changzhou 213001, China; 2 Department of Pharmacy, China Jiliang University, Hangzhou 310000, China

**Keywords:** circular RNAs, secondary structure prediction, folding stability, alternative structures, web server

## Abstract

Circular RNAs (circRNAs) are a novel class of non-coding RNA that, unlike linear RNAs, form a covalently closed loop without the 5′ and 3′ ends. Growing evidence shows that circular RNAs play important roles in life processes and have great potential implications in clinical and research fields. The accurate modeling of circRNAs structure and stability has far-reaching impact on our understanding of their functions and our ability to develop RNA-based therapeutics. The cRNAsp12 server offers a user-friendly web interface to predict circular RNA secondary structures and folding stabilities from the sequence. Through the helix-based landscape partitioning strategy, the server generates distinct ensembles of structures and predicts the minimal free energy structures for each ensemble with the recursive partition function calculation and backtracking algorithms. For structure predictions in the limited structural ensemble, the server also provides users with the option to set the structural constraints of forcing the base pairs and/or forcing the unpaired bases, such that only structures that meet the criteria are enumerated recursively.

## 1. Introduction

Most RNA molecules are linear. Circular RNAs (circRNAs) are a small but novel class of non-coding RNA that, unlike linear RNAs, form a covalently closed loop without the 5′ and 3′ ends. With the development of high-throughput sequencing technology and promising bioinformatics tools, emerging evidence indicates that circRNAs are stable, widely expressed, and play multifunctional roles in life processes, such as miRNA sponges, regulators of gene splicing and transcription, RNA-binding protein sponges and protein/peptide translators [1,2,3,4,5,6]. In consideration of their association with diseases [7,8,9,10,11,12,13], circRNAs have become a new hotspot in the clinical and research fields. Therefore, the accurate modeling of circRNAs annotation, structure determination and interaction analysis has a far-reaching impact on our understanding of their functions and our ability to develop RNA-based therapeutics [14,15,16,17,18,19,20,21,22].

Computational algorithms for predicting the secondary structures of circRNAs have been implemented in the Mfold [23,24] and Vienna RNA [25,26] package. Mfold predicts circRNA secondary structures from the “internal” and “external” linear subsequences separated by a base pair. It requires twice as much time and computer storage as the linear algorithm for a sequence of the same size. The Vienna RNA algorithm, furthermore, optimizes the memory requirements for the circRNA folding with the proper treatment for the exterior hairpin loops, internal loops, and multiloops within the “external” subsequence. It predicts circular secondary structures without additional memory requirements as a kind of “post-processing” of the linear structures.

Previous studies of the helix-based strategy for RNA folding kinetics prediction [27,28,29,30,31] implies that the landscape partitioning approach has the ability to predict alternative secondary structures for linear RNAs. Since the whole folding landscape is divided into discrete folding partitions through stable helices, each partition defines a structural ensemble mainly featured by the inclusion and exclusion of selected stable helices. Therefore, there is at least one helix difference between any two of them, making it better to address the conformational heterogeneity in RNAs [30,31]. Here, we apply the helix-based landscape partitioning strategy to develop a new model, cRNAsp12 (circular RNA structure prediction, from 1D to 2D), for predicting secondary structures of circRNAs. For a given folding temperature, the model uses the stable helical regions to define the main features of distinct structural ensembles in circular RNA folding landscape and determine the stable secondary structures of each ensemble with the corresponding constraints from the selected stable helices. By adding the structural constraints of forcing base pairs and/or forcing unpaired bases, furthermore, the model has the ability to predict circRNA secondary structures within the limited folding landscape. The cRNAsp12 model is freely accessible at the cRNAsp12 web server (http://xxulab.org.cn/crnasp12 accessed on 15 December 2022) for the prediction of circRNA secondary structures and folding stabilities from the given sequence.

## 2. Results

The cRNAsp12 web server is a user-friendly platform for the prediction of circular RNA secondary structures and folding stabilities. The computational time scales with the chain length *N* as O(*N*^3^) and the computer memory scales as O(*N*^2^). To avoid a long computational time, the current version of the cRNAsp12 server restricts the input sequence length up to 500 nts.

### 2.1. cRNAsp12 Input

**Figure 1:**
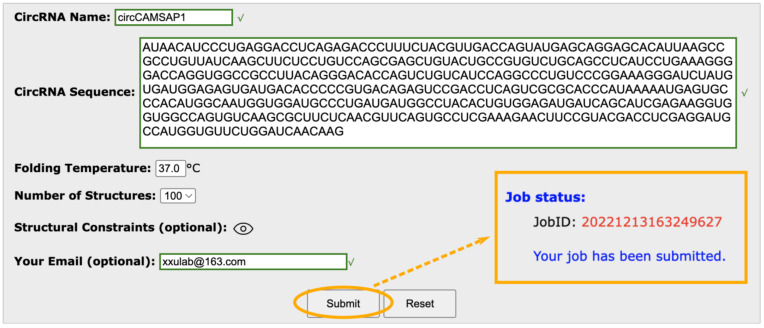
Snapshot of the input of the cRNAsp12 server. Once a calculation is submitted, a notification page containing the assigned job ID, as indicated by the dashed arrow, is displayed.

To predict the circular RNA secondary structures, the cRNAsp12 server requires users to input the circRNA sequence in plain text form (see the snapshot of a web server in Figure 1). The default folding temperature is 37 °C. Users have the option to change the temperature to other desired values. Since the model predicts alternative structures with the helix-based landscape partitioning strategy, users have the option to set the maximum number of predicted structures (five in default) from the drop-down list, such that the model selects saturated helices from the most stable to least stable ones until the number of partitions reaches the user-selected value. Once a calculation is submitted, a unique job ID is assigned and a notification page (see the snapshot of web server in Figure 1) containing job information, such as the circRNA name, sequence, folding temperature, e-mail address (if provided), structural constraints (if provided), and the assigned job ID, is displayed. If the e-mail address is provided during job submission, the cRNAsp12 web server automatically sends out an e-mail notification with the results attached once the calculation is completed.

**Figure 2:**
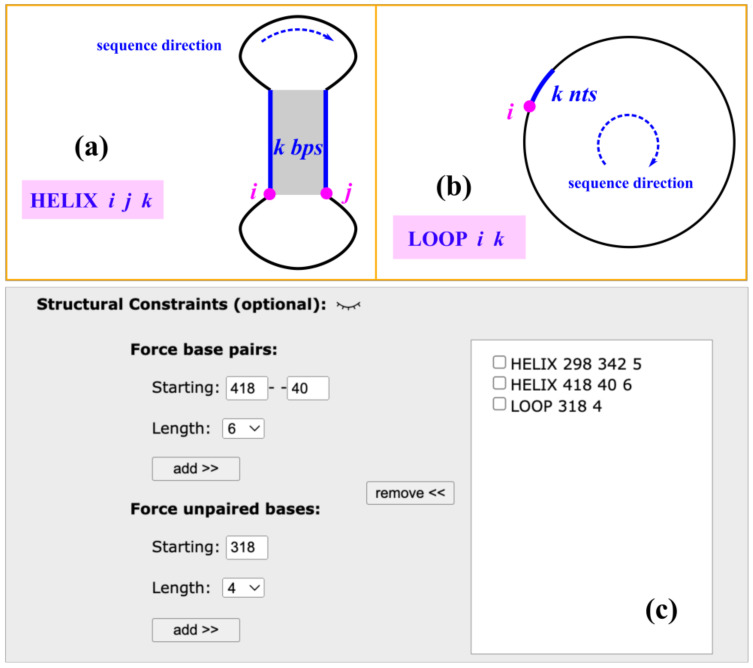
Structural constraints of forcing base pairs (HELIX *i j k*) in (**a**) and forcing unpaired bases (LOOP *i k*) in (**b**). (**c**) Example of setting the structural constraints for cRNAsp12 prediction. Here, *i* and *j* are the nucleotide numbering within the range of (1, *N*), with *N* as the length of the circRNA sequence, and *k* as the number of base pairs or unpaired bases.

For structure predictions in the limited (not full) structural ensemble, the server also provides users with the option to set the structural constraints of forcing the base pairs (HELIX *i j k*) and/or forcing the unpaired bases (LOOP *i k*), as shown in Figure 2a,b. For the forced base pairs (HELIX *i j k*), *i*–*j* is the sequence positions of starting base pair and k (bps) is the length of the forced helix. All forced base pairs should be canonical (A–U, G–C, and G–U). For the unpaired bases (LOOP *i k*), *i* is the sequence position of the starting nucleotide and *k* (nts) is the length of the forced loop. The values of *i* and *j* are in the range of (1, *N*) with *N* as the length of input circRNA sequence, and the value of *k* is selected from the drop-down list. As shown in Figure 2c, users can easily use the “add” and “remove” buttons to set constraints properly. It should be noted that improper settings of structural constraints, such as the overlapping or crossing between forced base pairs, may lead to no predictions.

### 2.2. cRNAsp12 Output

**Figure 3:**
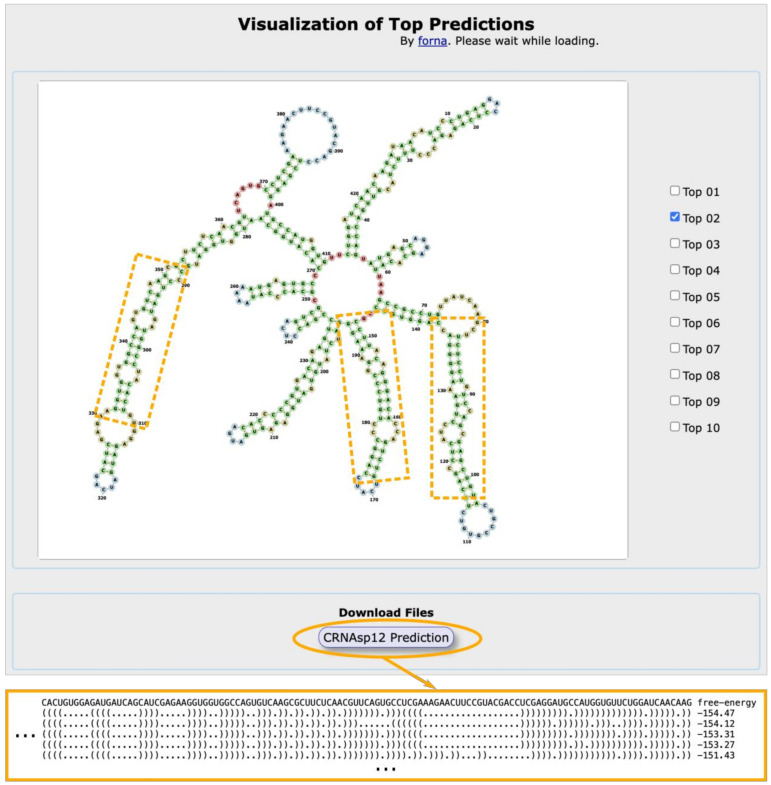
Snapshot of the output of the cRNAsp12 server. The model correctly predicts the three 16–26 bp dsRNAs, as indicated by the dashed rectangles, for the *CAMSAP1* circRNA.

By visiting the “Job Status” page; on the other hand, users have the option to retrieve the job status through the job ID (for one particular calculation) or e-mail address (for calculations submitted with the same e-mail address). There are three types of job status: “submitted”, “completed”, and “error”. For the completed jobs, users can visit the cRNAsp12 results page through the hyperlinks from the corresponding job IDs, as shown in Figure 3. In order to save storage space, the server keeps all the job files for only six months from the date of submission. However, the server may return no predictions for jobs with improper settings of structural constraints, whose statuses are marked as “error”.

The server provides the results of the predicted secondary structures (in dot-bracket notation) in text format for download. It also provides a *forna* javascript viewing container for the visualization of the cRNAsp12 predicted top-stable structures [32]. Figure 3 shows an example of cRNAsp12 prediction for the *CAMSAP1* circRNA with 425-nt in length (its sequence shown in Figure 1). The predicted secondary structures are ranked by their folding stabilities, i.e., the free energies in kcal/mol calculated with the base stacking and loop entropy energies.

### 2.3. Performance Benchmark

We used the dataset of 25 circRNAs with their sequence lengths ranging from 161 nts to 435 nts to benchmark the performance of the cRNAsp12 model. The detailed SHAPE-Map analysis and SHAPE-directed secondary structure modeling by RNAfold [25,26] reveal that those circRNAs tend to form 16–26 bp imperfect RNA duplexes and act as the inhibitors of double-stranded RNA (dsRNAs)-activated protein kinase (PKR) related to innate immunity [33]. Without integrating the SHAPE data, the cRNAsp12 model predicts their secondary structures at the folding temperature = 37 ℃.

As listed in Table 1, there are 46 16–26 bp dsRNAs in the SHAPE-directed secondary structures of 25 circRNAs, and cRNAsp12 correctly predicts the majority of them (36 out of 46). Specifically, all the dsRNAs in 19 circRNAs are correctly predicted (see Figure 3 and Figure S1). The dsRNAs in four circRNAs (*EPHB4*, *PVT1*, *FKBP8*, and *KIAA0368*) are partially predicted, while cRNAsp12 obtains the completely incorrectly predictions of dsRNAs for the *CCNB1* and *EZH2* circRNAs (see Figure S2). As in the example of the *CAMSAP1* circRNA, shown in Figure 3, cRNAsp12 correctly predicts the three 16–26 bp dsRNAs, as indicated by the dashed rectangles.

Moreover, we use the online servers of Mfold and RNAfold with the default parameters, except for the type of RNA sequence (circular), to predict the circRNA secondary structures for the above 25 tested cases. Similar to cRNAsp12, Mfold also correctly predicts the majority of them (32 out of 46). Since the RNAfold server only outputs the minimum free energy (MFE) structure for a given sequence, its prediction accuracy (RNAfold-alone without SHAPE) for the prediction of 16–26 bp dsRNAs is much lower than that of Mfold and cRNAsp12. As shown in Figure S2, most SHAPE-RNAfold predicted structures contain RNA secondary structural motifs with large-size loops. For example, the four-way junction of the SHAPE-RNAfold predicted CCNB1 circRNA has 34 unpaired nucleotides. Without the help of SHAPE data, the predicted structures of RNAfold, Mfold, and cRNAsp12 usually contain only the structural motifs with small-size loops. However, if the 16–26 bp dsRNAs contain stable helices, the helix-based landscape partitioning strategy for alternative structure prediction will more likely capture those dsRNAs.

## 3. Discussion

**Figure 4:**
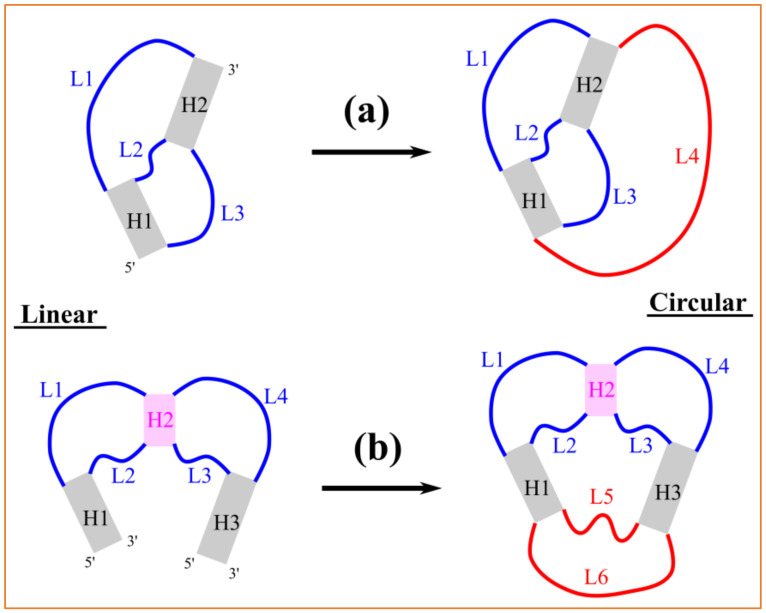
Extension of the H-PK and L-KISS motifs. (**a**) The H-PK motif of a linear RNA contains two helices and three loops (**left**). (**b**) The L-KISS motif of two linear RNAs contains three helices (H2 is the kissing helix) and four loops (**left**). For circular RNAs (**right**), the H-PK and L-KISS motifs need to be extended with the additional loops (marked in red).

RNAs can fold into structures with cross-linked base pairs, such as the pseudoknotted (H-PK), and hairpin–hairpin loop kissing (L-KISS) motifs shown in Figure 4. Because of the coupling effects between helices and loops, the corresponding energy parameters are mutually dependent on the sizes of loops and helices [34,35,36,37,38]. Due to the circular nature of circRNAs, the two above motifs need to be extended with additional loops, as shown in the red lines in Figure 4. The additional loops (L4 for H-PK, and L5 and L6 for L-KISS) are actually coupled with the existing helices and loops. As a result, the previously derived energy parameters from the Vfold model are no longer applicable. Therefore, the current version of the cRNAsp12 model can only treat circular RNA structures without cross-linked base pairs. Once the energy parameters are expanded, the model can be extended for the structure and stability prediction of complex circRNAs.

RNAs are negatively charged molecules. The ionic solution condition, such as ion concentration, size and charge, plays important roles in determining RNA thermal stability and folding kinetics [39,40,41,42]. The Turner parameters for the base stacking energies were experimentally measured with the fixed ionic solution condition (i.e., 1 M NaCl) [43]. However, there are currently no comprehensive energy parameters, similar to the Turner parameters, that have been experimentally measured in other ionic conditions. Modeling with such parameters should generally overestimate the helix stabilities for RNA structures in lower salt conditions. For RNA loop elements, on the other hand, there may be many sequence-dependent non-canonical base pairing interactions besides the loop entropy energies that contribute to the loop stabilities [44,45,46,47,48]. However, the available energy parameters (both experiment-measured and model-derived) are simply loop size-dependent. Effectively accounting for the loop sequence and ion effects on loop stability challenges the further development of models for the better prediction of RNA structures, stabilities and folding kinetics.

In recent years, machine learning (ML), especially deep learning, has made remarkable progresses in a wide range of fields, including RNA secondary structure prediction [49,50,51,52]. Due to the lack of sufficient data for circular RNAs, to our knowledge, currently available ML-based models are designed only for linear RNAs. Moreover, although ML-based methods have enabled us to more accurately predict RNA secondary structures, the estimation of their folding stabilities is also important for applications other than structure prediction. Hybrid methods that combine thermodynamic and ML-based approaches have been developed with the expectation to evaluate thermodynamic stability with high accuracy [53,54]. Therefore, with the further expansion of biological data, developing robust and explainable ML-based approaches with thermodynamic regularization would be an alternative and feasible means of accurately modeling circRNA secondary structures and stabilities.

## 4. Materials and Methods

**Figure 5:**
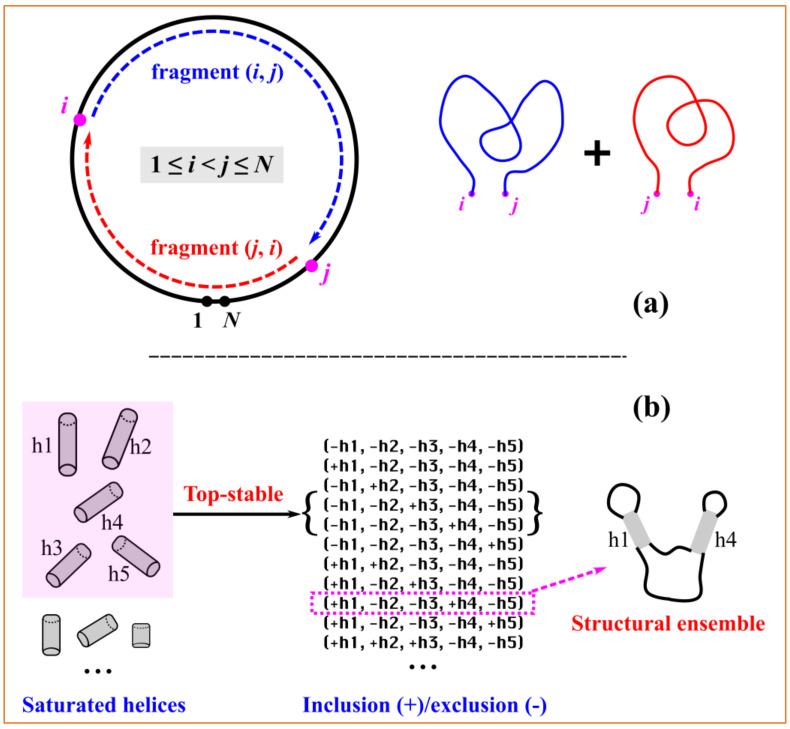
Algorithm of the cRNAsp12 model. (**a**) Two nucleotides are selected to divide a circular RNA sequence into two linear fragments. Secondary structures and folding stabilities of a circRNA are calculated by the additivity assumption characteristic of recursive algorithms. Here, *i* and *j* are the nucleotide numbering within the range of (1, *N*). (**b**) By selecting top-stable helices, the entire folding landscape is divided into distinct partitions. Each partition defines a structural ensemble featured by the inclusion/exclusion of helices.

Although the direction of numbering is uniquely defined, any consecutive numbering of the nucleotides beginning at an arbitrary point should be equivalent for circRNAs. By selecting any two (different) nucleotides, a circular sequence can be divided into two linear subsequences. For a given numbering of (1, *N*), with *N* as the length of the circRNA sequence, as shown in Figure 5a, the selected *i*th and *j*th nucleotides with 1≤ *i* < *j* ≤ *N* divide the entire circular sequence into two linear fragments, i.e., the “internal” fragment (*i*, *j*) and the “external” fragment (*j*, *i*) spanning over two pseudo-ends. The cRNAsp12 model combines the recursive partition function calculation with the backtracking algorithm to predict secondary structures for each linear fragment. The circRNA secondary structures and folding stabilities are calculated by the additivity assumption characteristic of recursive algorithms.

### 4.1. Energy Parameters

For the free energy-based RNA structure modeling, the predicted secondary structures, stabilities and folding kinetics could be sensitive to the choice of energy parameters. RNA secondary structures are defined by the base pairing patterns, which can be further divided into helices and loops. Based on the nearest-neighbor model, the total folding stability of an RNA secondary structure can be evaluated through the summation of the folding energies of individual helices and loop elements. For the helical regions, it is quite common to use the Turner parameters [43] to calculate the free energies from base stacks. For the RNA loop elements, such as the hairpin loops, internal/bulge loops, and multi-branched junctions, the cRNAsp12 model uses the Vfold-derived energy parameters to calculate the free energies from loop entropies, which has the advantage of accounting for chain connectivity, excluding the volume between helices and loops and the completeness of conformational ensemble [34,35,36,37,38].

### 4.2. Helix-Based Landscape Partitioning

For a given circRNA sequence, cRNAsp12 enumerates all the possible saturated helices formed by the canonical base pairs (A–U, G–C, and G–U), which cannot be further extended on either side through the canonical base pairs. As shown in Figure 2A, each helix is denoted by (*i*, *j*, *k*) with (*i*, *j*) the starting base pair and *k* is the number of canonical base pairs. If (*i* − 1, *j* + 1) and (*i* + *k*, *j* − *k*) are both noncanonical, then (*i*, *j*, *k*) is a saturated helix. The stability of each saturated helix is evaluated by the base stacking energies from the Turner parameters. The cRNAsp12 model ranks all the helices according to their free energies and selects top-stable helices to divide the entire folding landscape into discrete folding partitions. By enumerating all the possible helix combinations (inclusion/exclusion of selected top-stable helices) and deleting those with helix overlaps (nucleotides belonging to two or more helices) or crossing (involving the cross-linked base pairs) among included helices, a list of folding partitions featured by the selected helices is obtained. As shown the example in Figure 5b, five top-stable helices are selected and the partition of (+h1, −h2, −h3, +h4, and −h5) contains helices h1 and h4 and excludes helices h2, h3, and h5, representing a structural ensemble containing all the secondary structures with the base pairs from the included helices but without the base pairs from the excluded helices. The total number of partitions is determined by the size of the selected top-stable helix pool. The more the stable helices are selected, the larger the number of partitions, and the smaller the difference between partitions.

### 4.3. Alternative Structure Determination

Through the fragment-based strategy shown in Figure 5a and the recursive partition function calculation for linear RNAs, the cRNAsp12 model calculates the conditional partition function of each partition for the structural ensemble conditioned by the formation of the *i*–*j* base pair:

(1) $$Q_{partition}^{i-j}=Q_{fragment\;\left(i,j\right)}^{i-j}\cdot Q_{fragment\;\left(j,i\right)}^{j-i},$$

where $Q_{fragment\;\left(i,j\right)}^{i-j}$ and $Q_{fragment\;\left(j,i\right)}^{j-i}$ are the conditional partition functions of the fragments (*i*, *j*) and (*j*, *i*), respectively, calculated over the corresponding structural ensembles with the terminal nucleotides base-paired. By minimizing the free energy $G_{partition}^{i-j}=-k_{B}TlnQ_{partition}^{i-j}$ ($k_{B}$ is the Boltzmann constant and *T* is the folding temperature) over all the possible base pairs, the model determines the minimal free energy (MFE) structure and folding stability of each partition with the backtracking algorithm for the fragments of (*i*, *j*) and (*j*, *i*). The detailed algorithms of the recursive partition function calculation and backtracking for linear RNAs can be found in the published papers [55,56,57].

Therefore, the selected top-stable helices define the scaffolds of partitions, while all the less-stable (not selected) helices are sampled recursively during the structure enumeration process to characterize the shape of partition landscapes. Each partition is represented by the corresponding MFE structure. Through the helix-based landscape partitioning and MFE structure backtracking, cRNAsp12 predicts the alternative secondary structures of a given circRNA sequence.

## 5. Conclusions

Combining the helix-based landscape partitioning strategy, recursive partition function calculation and backtracking algorithms, we developed the cRNAsp12 software and web server to predict circular RNA secondary structures and folding stabilities from the sequence. Using stable helices to effectively divide the whole folding landscape into discrete structural ensembles, the helix-based folding model can better address the conformational heterogeneity in RNAs. Currently, the model can only treat circRNA secondary structures without the cross-linked base pairs. In fact, similar to linear RNAs, circular RNAs may fold into structures with long-range tertiary interactions, especially for long-size circRNAs. In a future work, cRNAsp12 will be continuously upgraded to treat circRNA structures including complex structural elements, such as the extended H-PK and L-KISS motifs.

Furthermore, the RNA environment in the solution is usually complicated, involving multiple surrounding molecules and ions. The interplay between them, especially the ion effects, plays important roles in determining RNA structures, stabilities, and functions. In future work, we will also add the effect of the ions to the enthalpy and entropy parameters for the loops and base stack formations to improve the performance of structure predictions. As an alternative future trend, developing the folding scores leant from the rich-parameterized weight parameters of ML-based models with thermodynamic integration for the modeling of circRNA secondary structures would also be a feasible direction for cRNAsp12.

## Figures and Tables

**Table 1:** Performance of the cRNAsp12 model.

Host Gene of circRNA	Length (nt)	No. of 16–26 bp dsRNAs			
		SHAPE-RNAfold ^1^	RNAfold ^2^	Mfold ^2^	cRNAsp12 ^2^
CAMSAP1	425	3	1	3	3
CCNB1	378	4	1	0	0
EPHB4	362	2	0	1	1
EZH2	253	1	0	1	0
FCHO2	268	2	1	2	2
FGFR1-1	179	1	0	1	1
PVT1	410	4	1	2	2
RELL1	434	2	0	1	2
SDHAF2	334	2	0	1	2
TBCD	389	3	3	3	3
UIMC1	397	2	0	1	2
ARID1B	286	2	0	0	2
CNN2	205	1	0	1	1
DHX34	218	1	0	1	1
FKBP8	259	2	2	2	1
KIAA0368	435	3	2	2	2
MBOAT2	224	1	1	1	1
PIP5K1C	249	1	1	1	1
PPP1CB	224	2	1	2	2
PROSC	220	1	1	1	1
PTK2	394	1	0	1	1
SLC22A23	259	2	2	2	2
SNHG4	161	1	1	1	1
TMEM181	324	1	1	1	1
VAPB	258	1	1	0	1
Average/Total	302	46	20	32	36

^1^ From Figure 4 of Ref [33]. ^2^ dsRNAs are considered correctly predicted ones if more than half of base pairs are correctly predicted.

## Data Availability

Not applicable.

## Supplementary Materials

The following supporting information can be downloaded at: https://www.mdpi.com/article/10.3390/ijms24043822/s1.
