# Supplementary material for: cRNAsp12 Web Server for the Prediction of Circular RNA Secondary Structures and Stabilities
Source: Int J Mol Sci. 2023 Feb 14;24(4):3822. doi: 10.3390/ijms24043822 (PMC9959564; doi:10.3390/ijms24043822)
Supplement: Supplementary file 1 [file ijms-24-03822-s001.zip › ijms-2131088-supplementary.pdf]

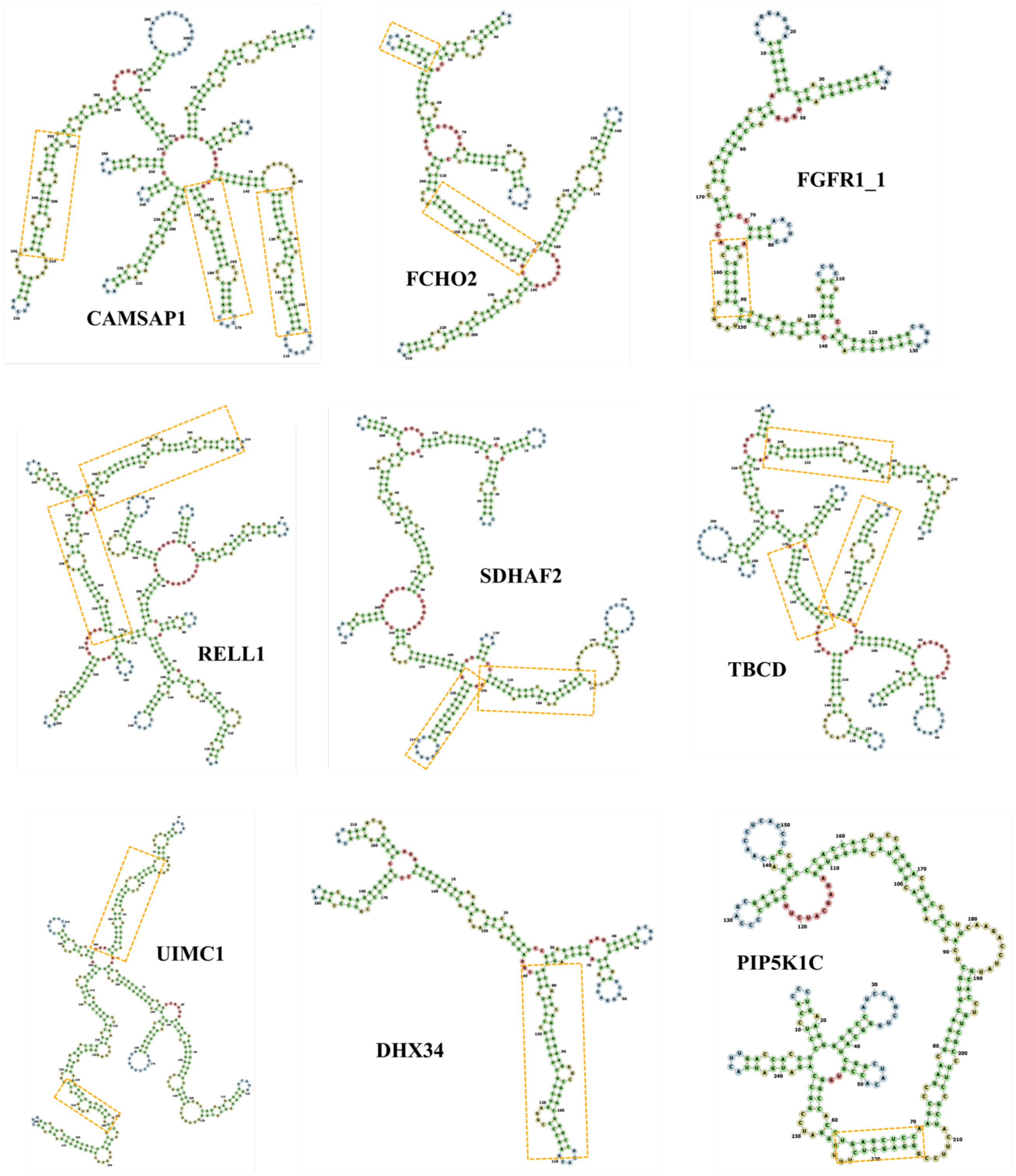

Figure S1. cRNAsp12 predictions with the 16-26 bp dsRNAs highlighted by the dashed rectangles.

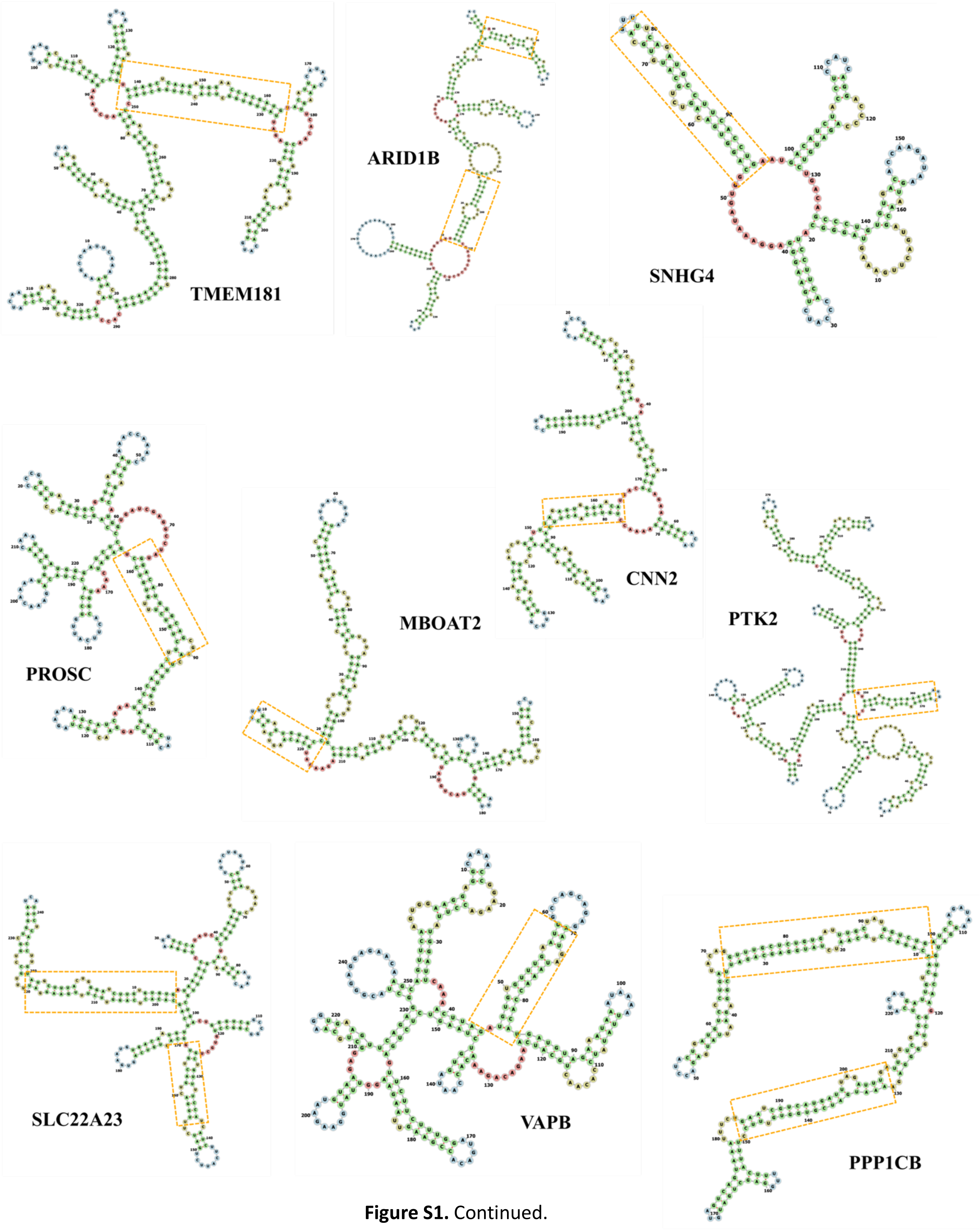

Figure S1. Continued.

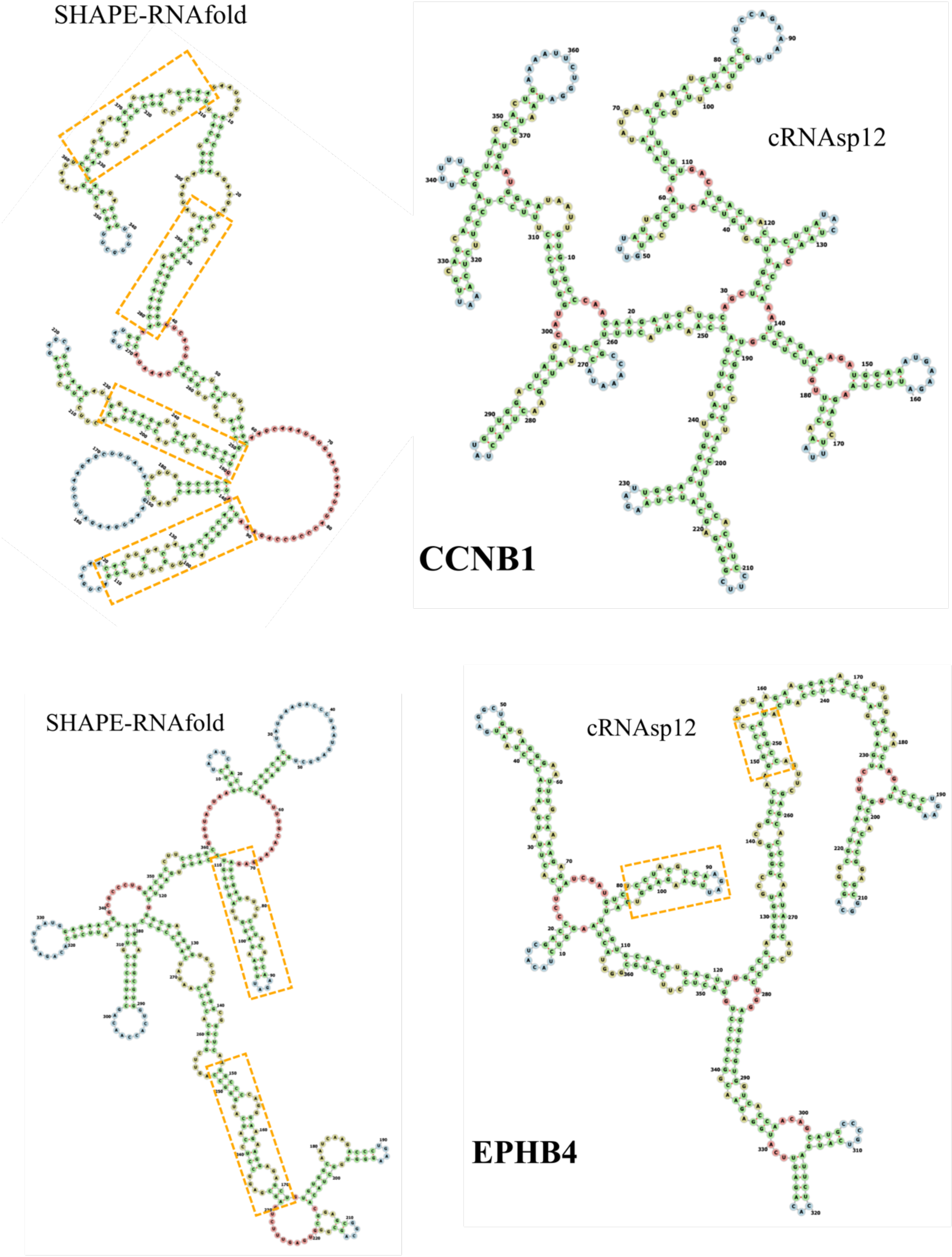

Figure S2. SHAPE-RNAfold and cRNAsp12 predictions with the 16-26 bp dsRNAs highlighted by the dashed rectangles.

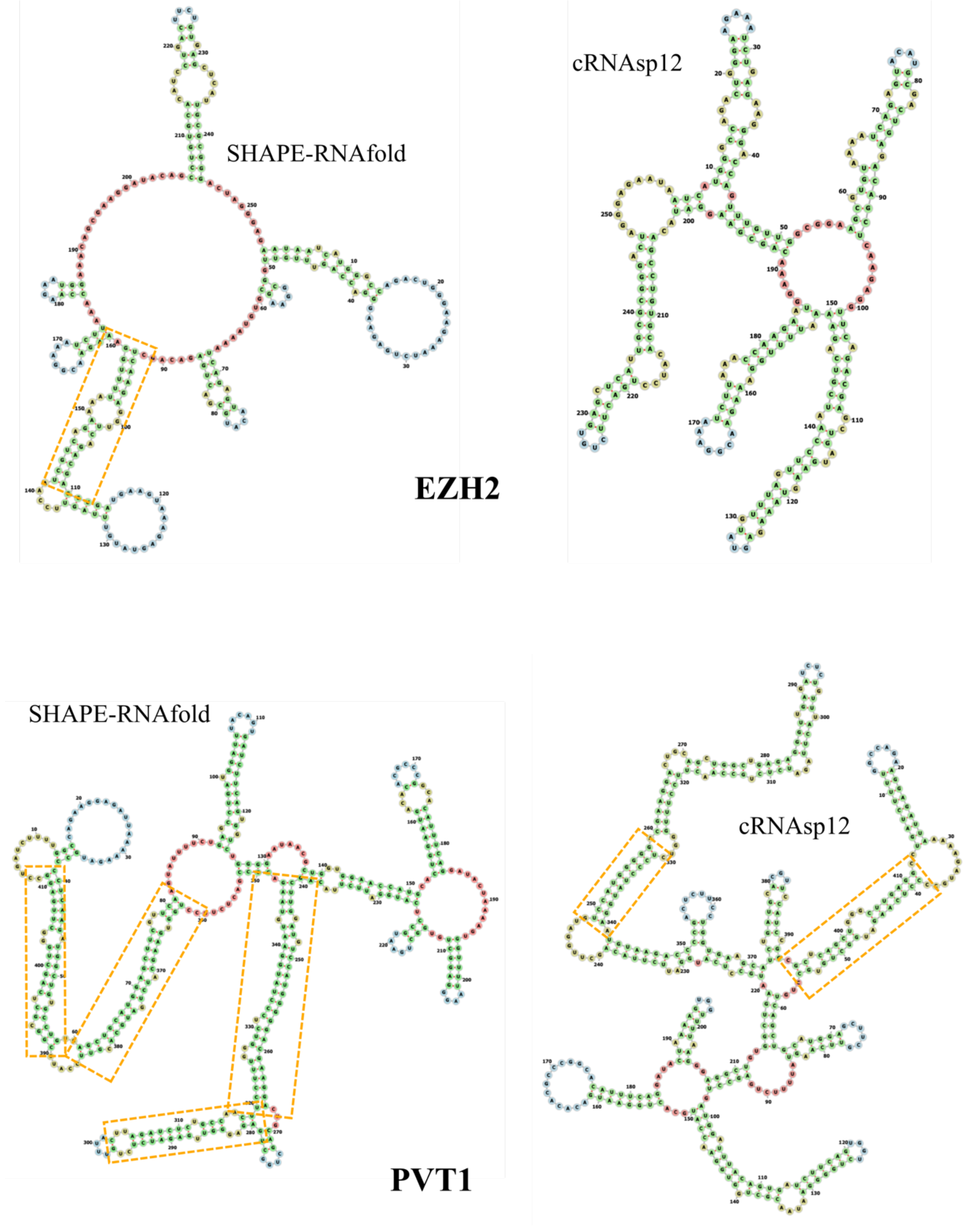

Figure S2. Continued.

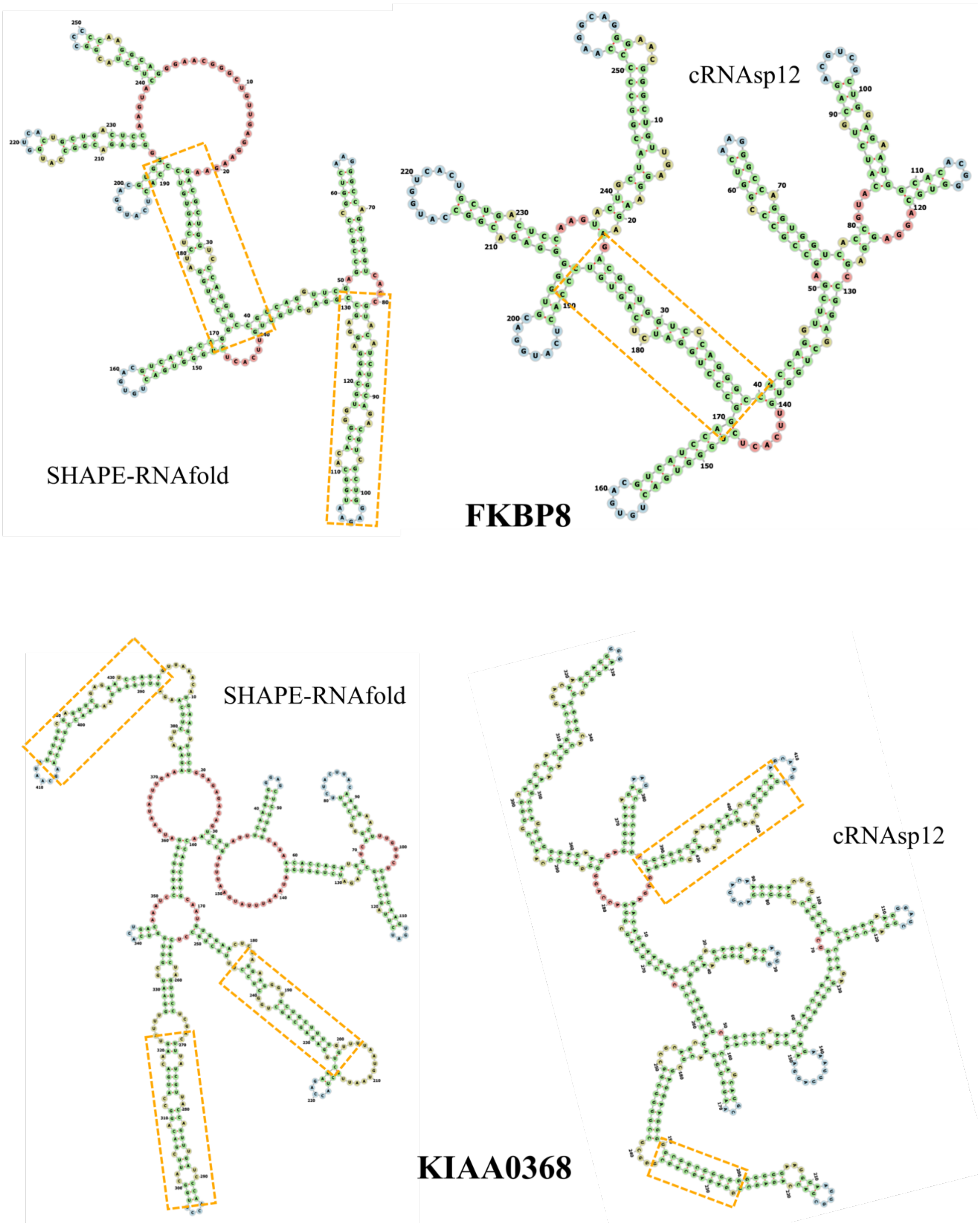

Figure S2. Continued.
